# DBT Masses Automatic Segmentation Using U-Net Neural Networks

**DOI:** 10.1155/2020/7156165

**Published:** 2020-01-28

**Authors:** Xiaobo Lai, Weiji Yang, Ruipeng Li

**Affiliations:** ^1^College of Medical Technology, Zhejiang Chinese Medical University, Hangzhou 310053, China; ^2^College of Life Science, Zhejiang Chinese Medical University, Hangzhou 310053, China; ^3^Hangzhou Third People's Hospital, Hangzhou 310009, China

## Abstract

To improve the automatic segmentation accuracy of breast masses in digital breast tomosynthesis (DBT) images, we propose a DBT mass automatic segmentation algorithm by using a U-Net architecture. Firstly, to suppress the background tissue noise and enhance the contrast of the mass candidate regions, after the top-hat transform of DBT images, a constraint matrix is constructed and multiplied with the DBT image. Secondly, an efficient U-Net neural network is built and image patches are extracted before data augmentation to establish the training dataset to train the U-Net model. And then the presegmentation of the DBT tumors is implemented, which initially classifies per pixel into two different types of labels. Finally, all regions smaller than 50 voxels considered as false positives are removed, and the median filter smoothes the mass boundaries to obtain the final segmentation results. The proposed method can effectively improve the performance in the automatic segmentation of the masses in DBT images. Using the detection Accuracy (Acc), Sensitivity (Sen), Specificity (Spe), and area under the curve (AUC) as evaluation indexes, the Acc, Sen, Spe, and AUC for DBT mass segmentation in the entire experimental dataset is 0.871, 0.869, 0.882, and 0.859, respectively. Our proposed U-Net-based DBT mass automatic segmentation system obtains promising results, which is superior to some classical architectures, and may be expected to have clinical application prospects.

## 1. Introduction

Breast cancer is the most common malignant tumor of breast epithelial tissue in women, which seriously threatens the physical and mental health of patients [1]. In recent years, breast cancer has become a significant public health problem in today's society with a rising incidence and the younger incidence groups. Early diagnosis and treatment can effectively reduce the mortality and improve the quality of life of patients [2, 3]. Digital breast tomosynthesis (DBT) is a new 3D tomography method for breast cancer screening. It can reconstruct a small amount of low-dose mammographic images from a limited angle into three-dimensional mammographic images and can better detect some small hidden lesions [4]. Compared with traditional mammography, although DBT has improved the sensitivity of detecting breast masses, it has also significantly increased the amount of interpretation data for radiologists, which is time consuming and poor repeatability [5–7]. If the computer can automatically detect breast masses in DBT images, it can not only reduce the review time for radiologists but also effectively reduce the misjudgment caused by excessive fatigue. Therefore, automatic segmentation of breast masses in DBT images is of great clinical value in assistant screening, early diagnosis, and preoperative localization of breast cancer [8–10].

Automatic segmentation of breast masses in DBT images is a challenging task because the signal-to-noise ratio of two-dimensional slice images of DBT is not high and the significance of breast masses is not strong [11]. Although it is difficult to achieve precise automatic segmentation of DBT tumors, many scientists have made fruitful explorations on automatic segmentation of DBT tumors in recent years because of its great significance in assistant diagnosis and treatment [12]. These studies can be roughly divided into two categories [13]. One is breast mass detection based on DBT reconstructed slice images. Reiser et al. [14] proposed a breast mass detection computer-aided diagnosis (CAD) system, which uses a radial gradient index to detect and segment suspicious lesions in DBT reconstructed images. The experimental results show that incorporating vertical direction information does not improve the performance of gradient-based classifiers, but it can improve the performance of shape-based classifiers. In another early study, Chan et al. [15] also proposed a method for automatic detection of breast masses, including DBT reconstruction image interpolation to obtain cubic pixels, gradient field analysis to determine suspicious regions, three-dimensional region growth segmentation, and feature analysis, with the sensitivity of 85%. Then, Chan et al. [16] studied the relationship between the number of projected images and the dose used to obtain DBT images and the performance of the algorithm and used a set of 21 two-dimensional projected images or 11 reconstructed slice images to test. Another is breast mass detection based on the two-dimensional projection image of DBT. van Schie et al. [17] proposed an automatic detection method for breast masses, using a mammography image library to train classifiers. To optimize and make the technique suitable for DBT images, tomographic images were generated from reconstructed volume images for analysis. Palma et al. [18] constructed a system of automatic detection of breast masses in DBT reconstruction images by using fuzzy theory and antagonistic reasoning method. Kim et al. [19] studied the influence of the saliency of reconstructed slice images on the detection performance of breast masses in DBT reconstructed images and proposed an automatic detection method of breast masses based on the saliency of reconstructed slice images by DBT. In addition, some researchers fused the information of projection images and reconstructed images to detect DBT tumors. A hybrid method of two-dimensional and three-dimensional images was used to segment DBT masses with projection image and reconstructed image information [20].

Over the last few years, convolutional neural network (CNN) based on deep learning has become a research hotspot in the field of computer vision because of its strong ability to express image features [21, 22]. It has achieved fruitful results in image recognition and classification, target detection, and other fields. Elboushaki et al. validated that the CNN model can recognize fine mammographic features [23]. Vigueras-Guillen et al. first proposed a full-convolution network for semantic segmentation, replacing the conventional full-connection layer in the CNN with the convolution layer to obtain a rough label graph and then using the deconvolution layer to sample the rough label graph to achieve the classification results of each pixel [24]. Ciresan et al. used patches of 101 × 101 pixels to train a CNN for mitosis detection in breast cancer histology images, who won the ICPR 2012 Mitosis Detection Contest with F1-score of 0.782 [25]. Zhang et al. proposed a new FCN-like structure, U-net, for Bio-cell image segmentation [26]. This method has attracted considerable attention in the medical field because the U-net architecture supports a small amount of the data training model and fast image segmentation with the trained model can be achieved. At present, this method has been applied to many different tasks and also achieved excellent results, such as image segmentation and image conversion. [27, 28].

In this work, we propose an efficient DBT masses automatic segmentation algorithm by using a U-Net architecture, which works with only weakly human-annotated mass masks. To suppress the background tissue noise and enhance the contrast of the mass candidate regions, we construct a constraint matrix, which is multiplied with the DBT image after the top-hat transform. A U-Net architecture is built, and image patches are extracted before data augmentation. Then, the presegmentation of the breast tumors in DBT images is implemented. All regions smaller than 50 voxels considered as false positives are removed and the final segmentation results are obtained after the median filter smoothes the mass boundaries. The proposed method can effectively improve the performance in the automatic segmentation of the masses in DBT images. The architecture is developed and evaluated with the DBT images database prepared by a neuroradiologist in our research team. Experimental results tested on the DBT database indicate that the presented DBT mass CAD architecture achieves the high level of segmentation. To our knowledge, this is the first DBT study to employ the U-Net framework to segment the masses in DBT images automatically.

The remaining sections are organized as follows. In Section 2, the proposed method is presented. The database used for evaluation is detailed and results are presented and discussed in Section 3. Finally, the main conclusions are presented in Section 4.

## 2. Methods

**Figure 1:**
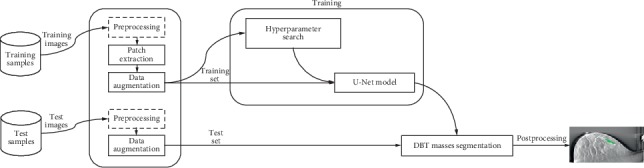
Overview of the presented method.

Our proposed approach consists of six main stages: DBT image preprocessing, patch extraction, data augmentation, voting scheme fusion, segmentation via the U-Net architecture, and postprocessing. An overview of our presented architecture is illustrated in Figure 1.

### 2.1. DBT Image Preprocessing

Usually, the random distribution of X-ray photons in mammography or DBT images will seriously affect the quality of breast images. However, as the average photon number (X-ray dose) increases, the noise will gradually decrease. For a typical DBT system, radiation exposure is an important factor to avoid the risk of radiation-induced cancer. Therefore, the low radiation dose is often used when creating the tomosynthesis images and the total radiation dose of DBT is slightly higher than that of standard mammography. In theory, the typical DBT images usually contain Poisson distribution noise. To address this issue, a top-hat transform is applied to enhance the contrast between candidate tumor location regions and background tissues, which is defined as

(1) $$\begin{array}{c} I_{\text{top}-\text{hat}}=I-\min\left(\left(I\cdot s_{c}\right)\circ s_{o};I\right), \end{array}$$

where *I* denotes the preprocessed image, · represents the morphological “closed” operation, ∘ denotes the morphological “open” operation, and *s*_*c*_ and *s*_*o*_ are the disc structure elements. Also, to enhance candidate location regions and suppress the background tissues, a constraint matrix we constructed is multiplied with the image matrix. The constraint matrix is generated by an isotropic radial basis function centered on the candidate location region with a variance *σ*^2^ (*σ* is 5 mm). It can be detailed that randomly selected one mark location *x*_*r*_ from all locations *x* in one image view, remove *x*_*c*_ from *x*, where

(2) $$\begin{array}{c} x_{c}=\left\{x_{i}\left|\left\|x_{i}-x_{r}\right\|\right.\leq 5,\;x_{i}\in x\right\}. \end{array}$$

**Figure 2:**
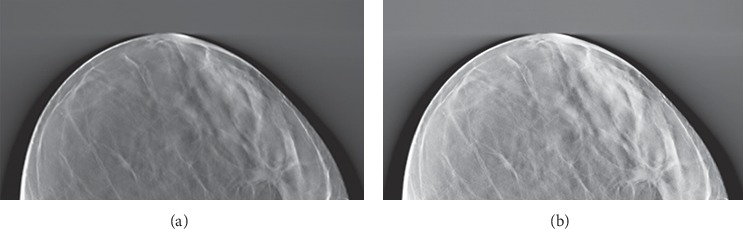
Preprocessing effects. (a) Original image and (b) preprocessed image.

Figure 2 shows the DBT image preprocessing effects, where Figure 2(a) is the original image and Figure 2(b) is the preprocessed image.

### 2.2. Image Patch Generation

Generally, it is straightforward to train the proposed U-Net directly by using the image patches extracted from the DBT mass regions because we have location information of the masses in the training and testing image sets. However, the available dataset has a small number of examples as compared to other U-net classification problems, and the direct use of whole images would most likely result in overfitting. This can be addressed by splitting images into patches which increases dataset complexity and dimension. In fact, nonmass regions can also provide some useful information for the breast mass segmentation task. In our model, the input to the U-Net architecture is a two-dimensional array, of shape height × width, since it consists of a two-dimensional patch of width × height voxels. The two-dimensional patches are taken along the *x*-*y* axis, also called the axial plane in anatomy. To avoid overfitting, we can extract the image patches from mass and nonmass regions to augment the training data. That is, we use the image patches extracted from the nonmass regions as additional negative samples for the U-Net architecture training, to help the proposed model to distinguish confounding regions from DBT masses.

In addition, the training data has to be balanced; that is, the same number of examples for each class should be included in the training data, which is to ensure that the U-Net model can generalize well. However, the number of pixels in the mass regions is significantly less than that in the nonmass regions, which leads to a severe class imbalance problem. To address this issue, we randomly resample at each epoch the same number of patches for each class from all possible patches for that class.

### 2.3. U-Net Architecture

**Figure 3:**
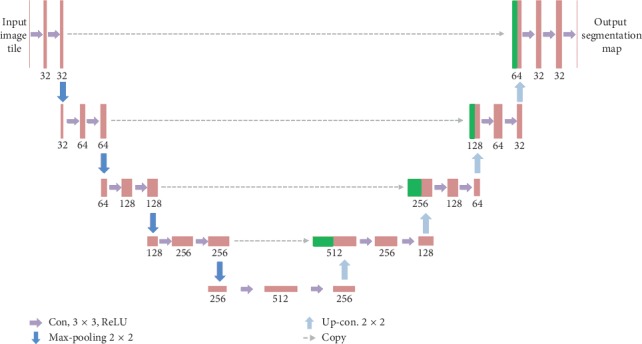
Proposed DBT mass segmentation U-Net architecture.

In this part, we will briefly introduce the architecture of the proposed typical U-Net model and its application to our DBT mass CAD system. We perform an end-to-end pixel-wise segmentation via a U-Net model. As shown in Figure 3, we illustrate the framework of our model. The proposed U-Net-based DBT mass segmentation architecture is mainly composed of a contracting path in the left side and an expansive path in the right side. The contraction path in the model follows the typical structure of the convolution network, including two 3 × 3 convolutions applied repeatedly, each convolution is followed by a rectified linear unit (ReLU) and a 2 × 2 max-pooling operation with stride 2, which is used for down sampling. In each of the down sampling step, we double the number of feature channels. Each step in the expansive path includes the up sampling of the feature map, followed by a 2 × 2 convolution (“up convolution”), which halves the number of feature channels, cascades them with the corresponding cropped feature map in the contraction path, and two 3 × 3 convolutions, each of which is followed by a ReLU. Because every convolution will lose the boundary pixels of the image, the image must be cropped. In the last layer of the network, a 1 × 1 convolution is used to map the characteristic vector of each 64 components to the required class number. In total, there are 23 convolutional layers in our proposed U-Net architecture.

### 2.4. Training Procedure

During the training process, we have balanced the training data by randomly resampling at each epoch the same number of patches for each class from all possible patches for that class. However, it is worth noting that the mass detection task still has a class-imbalance problem, where the number of positive samples (i.e., pixels in mass regions) is much less than the number of negative samples (i.e., pixels in nonmass regions). Hence, in our study, the proposed U-Net model uses the *F*_*β*_-measure as the cost function, rather than the cross-entropy-based or the quadratic cost function. Denote *S* and *T* as the predicted heatmap and the ground truth heatmap, respectively. Let *M* represent the number of elements (pixels) in *S* and *T*, and the *F*_*β*_-measure based loss function is defined as

(3) $$\begin{array}{c} F_{\beta }\left(S,T\right)=\frac{\left(1+\beta^{2}\right)\sum_{i=1}^{M}s_{i}t_{i}}{\sum_{i=1}^{M}s_{i}+\sum_{i=1}^{M}t_{i}}, \end{array}$$

where *s*_*i*_ is the *i*th element of the predicted heatmap and *t*_*i*_ is the *i*th element of the ground truth heatmap. In our study, we set *β*=1.

In the training procedure, the input images and their corresponding segmentation heatmaps are used to train the U-Net model with the stochastic gradient descent. Besides that, we applied the Adaptive Moment Estimation Method (Adam) [29] which is a stochastic gradient descent method that computes adaptive learning rates for each parameter to minimize the *F*_*β*_-measure-based loss function. The Adam optimizer parameters in our proposed U-Net architecture are set as learning rate = 0.0002 and the maximum number of epochs = 150. We adopted a Xavier normal heuristic [30] to initialize kernel weights in our study, which allowed us to maintain the gradients in controlled levels and thus prevent gradient vanishing during back-propagation. The biases are all initialized to 0. Because of the unpadded convolutions, the size of the output image is smaller than that of the input image by a constant border width. In order to minimize the overhead and maximize the use of GPU memory, we tend to make large input tiles over a large batch size, and therefore reduce the batch to a single image. Therefore, we apply a high momentum to make a large number of the previously seen training samples to determine the update in the current optimization step.

### 2.5. DBT Data Augmentation

To improve the performance of U-Net, we need to extend the data to generate more training data from the original data. In typical applications of the U-Net neural network for image processing and computer vision tasks, translations and rotations are used. In this study, the data consists entirely of two-dimensional patches. Thus, translation cannot be used as it would result in a different patch, with a possibly different label. However, using rotations of the patches might give some performance improvements. Therefore, we perform the rotations by using angles multiple of 90°.

### 2.6. Voting Scheme

Every test DBT image is first split into a set of patches, and for each patch a probabilistic prediction *p*_*i*_ ∈ [0,1] is made using the U-Net model. These predictions are then fused into the final image label using one of the following three voting schemes. The first three (Majority voting, Maximum probability, and Sum of probabilities) are also used and compared [31], whereas the other one (Connectivity) is proposed by us. Our motivation behind this voting schemes is to reinforce spatial consistency between votes of patches because in reality the true image label is likely to be assigned based on the structure of a particular connected region of the tissue rather than on many disconnected bits. In particular, if patches that vote for the same label are adjacent to each other, then this should be a more significant vote than if they are separated. The precise definitions follow.

#### 2.6.1. Majority Voting

Let us define the number of patches that vote for the class label *k* as

(4) $$\begin{array}{c} v_{k}=\underset{i\in P}{\sum }\prod \left(l_{i}=k\right), \end{array}$$

where *l*_*i*_ is the class label of patch *i*. The image label $\tilde{k}$ is then selected as the most common patch label by

(5) $$\begin{array}{c} \tilde{k}=\arg\underset{k\in K}{\max}\left(v_{k}\right). \end{array}$$

#### 2.6.2. Maximum Probability

The patch with the highest-class probability decides the image class label as

(6) $$\begin{array}{c} \tilde{k}=\arg\underset{k\in K}{\max}\left(\underset{i\in P}{\max}\left(p_{i}\left[k\right]\right)\right), \end{array}$$

where *p*_*i*_[*k*]=*p* (patch *i*∈class *k*).

#### 2.6.3. Sum of Probabilities

The patch class probabilities are summed and the class with the largest sum is chosen as

(7) $$\begin{array}{c} \tilde{k}=\arg\underset{k\in K}{\max}\left(\underset{i\in P}{\sum }p_{i}\left[k\right]\right). \end{array}$$

#### 2.6.4. Connectivity

This method is based on counting the number *c*_*k*_ of connections for each class *k*, where the connection means that two adjacent patches have the same class label *k*. These counts are calculated as

(8) $$\begin{array}{c} c_{k}=\underset{i\in P}{\sum }\underset{j\in P_{i}}{\sum }\prod \left(l_{i}=l_{j}\right), \end{array}$$

where *P*_*i*_ is the set of patches adjacent to patch *i*, including the patches along diagonals. The obtained counts are then used to weight the class votes *v*_*k*_ as

(9) $$\begin{array}{c} \tilde{k}=\arg\underset{k\in K}{\max}\left(\frac{c_{k}v_{k}}{\sum_{k^{\prime }\in K}c_{k^{\prime }}}\right)=\arg\underset{k\in K}{\max}\left(c_{k}v_{k}\right). \end{array}$$

### 2.7. Segmentation Postprocessing

Some small clusters may be mistakenly classified as the DBT masses. To deal with this issue, we impose volumetric constrains by removing clusters in the segmentation obtained by the U-Net that are less than 50 voxels in volumes.

## 3. Experimental Details

### 3.1. Materials

The benchmarking clinical DBT images used are collected at Zhejiang Chinese Medical University Affiliated Guangxing Hospital and Zhejiang Provincial Hospital of Traditional Chinese Medicine (TCM) with Institutional Review Board (IRB) approval. Every DBT image is produced by low dose exposure, where the total shot dose should be within the range of a regular mammogram dose. DBT cases are acquired in mediolateral oblique (MLO) and craniocaudal (CC) views (Siemens Mammomat Inspiration DBT system) using a total tomographic angular range of 60° with a 5° increment of rotation and 12 projection views. The DBTs are reconstructed to the images with 1 mm slice spacing by using the simultaneous algebraic reconstruction technique (SART). We convert the images into TIFF stack/slices and used data in JPEG format. Depending on the thickness of the breast, each DBT volume provides between 50 and 80 2D slices with a resolution of 1200 ± 901 pixels, which are saved in the JPEG format.

The database consists of 87 DBT volumes and 3960 2D X-ray images slices. Among these cases of breast cancer patients, 29 are malignant and 42 are benign (absolute healthy). The noncancerous DBT volumes are collected from the left and right breasts of 23 patients without early signs of breast cancer. The cohort of cancerous cases is annotated by two experienced radiologists with a 2D bounding box for DBT.

### 3.2. Experiments Design

Our purpose is to evaluate three scenarios that reflect common practices in research and evaluation of the DBT mass segmentation with the U-Net model:

- The data used for model training and for model testing are from the same hospital
- The data used for model training and for model testing are from a different hospital
- The data used for model training are from the same institution as the data for model testing are enriched by additional data coming from a different hospital resulting in an increased size of the training dataset

We use the following way to simulate three scenarios with 5-fold crossvalidation. First, we number the DBT slice images serially for each hospital. Within a fold, the serial numbers for training and test set are obtained. To automatically segment the DBT mass in the test set, we develop three U-Net models as follows. (i) The first U-Net model is trained on the DBT images from the same hospital using the serial numbers in the training set. (ii) The second U-Net model is trained on the DBT images using the training set serial numbers from the other dataset. In this way, we use the same number of DBT images used for training the U-Net model. (iii) The third U-Net model is built using all the DBT images used in (i) and (ii) such that DBT images from both hospitals are used. Hence, we use six types of train-test combinations: (a) train on hospital 1 (denoted as H1), test on H1, (b) train on hospital 2 (denoted as H2), test on H2, (c) train on H1, test on H2, (d) train on H2, test on H1, (e) train on (H1 + H2), test on H1, and (f) train on (H1 + H2), test on H2. Please note that the DBT images segmented by a U-Net model are never present in the training set for that U-Net model. For each DBT image under test, we classify each voxel into one of the two classes (nonmass region and mass region).

### 3.3. Evaluation Metrics

To enable comparison with other state-of-the-art works, we used three metrics commonly found in the literature: Accuracy (Acc), Sensitivity (Sen), and Specificity (Spe) as evaluation of classification results. Acc refers to the ratio of the number of pixels correctly segmented to the number of total pixels in the image, Sen refers to the probability of a positive test among the subjects with the condition, and Spe refers to the probability of a negative test among the subjects without the condition. The DBT masses segmentation evaluation metrics are defined as follows:

(10) $$\begin{array}{c} \text{Sen}=\frac{\text{TP}}{\text{TP}+\text{FN}},\\ \text{Spe}=\frac{\text{TN}}{\text{TN}+\text{FP}},\\ \text{Acc}=\frac{\text{TP}+\text{TN}}{\text{TP}+\text{TN}+\text{FP}+\text{FN}}, \end{array}$$

where TP, FP, TN, and FN denote true positive, false positive, true negative, and false negative, respectively.

Additionally, to evaluate the robustness of our proposed U-Net model, the receiver operating characteristic (ROC) curve and the average area under the curve (AUC) are calculated and compared.

The proposed approach is implemented in Python using the machine learning library Keras. The training and test experiments are performed using the cloud computing service PAI-DSW provided by Ali. Specifically, we use the runtime platform processor of Intel (R) Core (TM) i7-6800K CPU @ 3.40 GHz, 32 GB RAM, Nvidia GeForce RTX 2080 Ti, 64-bit Windows 10. The presented figures are produced using the plotting library matplotlib. All the parameters are set according to our preexperimental study, and it takes about 56 minutes to learn the parameters. An example code is shown below:

  import numpy as np
  import configparser
  import matplotlib as plt
  import os
  from keras.models import Model
  from keras.layers import Input, concatenate, Conv2D, MaxPooling2D, UpSampling2D, Reshape, core, Dropout
  from keras.optimizers import Adam
  from keras.callbacks import ModelCheckpoint, LearningRateScheduler
  from keras import backend as K
  from keras.optimizers import SGD
  from lib.help_functions import ∗

### 3.4. Results and Analysis

**Figure 4:**
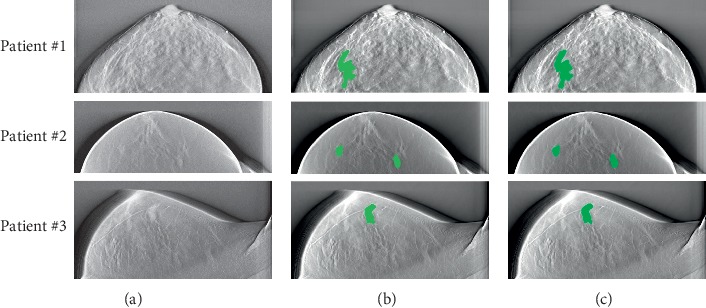
Illustration of predicted DBT mass of three patients. (a) Original image. (b) Segmented image. (c) Ground truth.

To assess the segmentation performance of our proposed method based on U-Net, we evaluated the overlap between the proposed DBT mass labels and the ground truth (GT).  Figure 4 showcases example results of the DBT mass automatic segmentation with the U-Net model trained and tested on the images (patient #1, patient #2, and patient #3) from the same hospital. The 1st row shows DBT original images for patient #1, patient #2, and patient #3, the 2nd row shows respective images segmented with our proposed U-Net architecture, and the 3rd row shows the same images segmented manually. The results indicate that our proposed U-Net model has high agreement between the generated results and the provided labels, and it takes about 132 seconds to label a sample.

Table 1 presents the performance of various voting schemes to assign final image labels. We can see that when the U-Net model is trained on the DBT images comes from the same hospital and tested on the DBT images comes from the same hospital, the maximum probability achieves best accuracy, which highlights the importance of a good voting scheme, namely, maximum probability. Contrary to my expectations, the method connectivity, whose aim is to reinforce consensus between spatially close patches, does not provide better results and scored the same as the majority voting. This may imply that having such spatial constraints is not relevant to this segmentation problem.

Table 2 shows the average Sensitivity (Sen) values of the DBT mass automatic segmentation by training-testing within the same hospital, across hospitals, AND with both hospitals using the maximum probability voting scheme. It can be found that the performance of the DBT mass automatic segmentation based on the proposed U-Net model significantly decreases when the proposed U-Net model is trained on data comes from a different hospital (Sen = 0.83 ± 0.015 for H1 and Sen = 0.85 ± 0.013 for H2) as compared with when it is trained with the data comes from the same hospital (Sen = 0.88 ± 0.009 for H1 and Sen = 0.89 ± 0.021 for H2).

Similar to Sensitivity (Sen), Specificity (Spe) shows that training the U-Net model on different hospital DBT images decreases the performance (Spe = 0.86 ± 0.009 for H1 and Spe = 0.87 ± 0.013 for H2) compared with training on the same hospital dataset (Spe = 0.89 ± 0.011 for H1 and Spe = 0.89 ± 0.017 for H2). All results using Spe are presented in Table 3. Accuracy (Acc) has been used as another metric of evaluation, as shown in Table 4. We find that when the proposed U-Net model is trained on different hospital DBT images, the Acc values are less than 0.87. However, when the proposed U-Net model is trained on the same or both hospitals DBT images, the Acc values are always greater than 0.87. Specifically, low Sen and high Acc can be obtained (Sen = 0.78 ± 0.015 and Acc = 0.86 ± 0.009) while training on H2 and testing on H1, which indicates that many FN are present, and high Sen and low Acc are obtained (Sen = 0.86 ± 0.013 and Acc = 0.79 ± 0.015) while training on H1 and testing on H2, which indicates many FN are present. For training and testing on the same hospital DBT images, both Sen and Acc are greater than 0.88 (Sen = 0.88 ± 0.009 and Acc = 0.88 ± 0.011 for H1) and (Sen = 0.89 ± 0.021 and Acc = 0.89 ± 0.017 for H2). Similar to training/testing on the same hospital, high Sen and Acc are obtained while training on both hospitals DBT images (Sen = 0.87 ± 0.011 and Acc = 0.86 ± 0.021 for H1) and (Sen = 0.88 ± 0.015 and Acc = 0.87 ± 0.019 for H2).

### 3.5. Discussion

To validate our U-Net CAD framework, we combine the two DBT image datasets into a bigger dataset, and the combination is denoted as the entire dataset. We compare the performance of various methods on automated DBT mass detection at aspects of the classifier used, DBT dataset size, Sen, Acc, and AUC in Table 5, our network achieve quite a competitive result than some of them. Among these models, we will discuss in detail the research works by Kim et al. [31], Fotin et al. [32], and Samala et al. [33], which applied deep learning methods to DBT mass detection and segmentation. Their works evaluated the DBT mass automatic segmentation CAD frameworks, which are based on both hand-crafted feature and deep convolutional neural network (DCNN)-based models. Samala et al. [33] proposed a DCNN architecture consists of four convolutional layers and three fully connected layers. Firstly, the DCNN model is trained on large-scale 2D mammography dataset, then the first three convolutional layers weights are frozen, and the rest of which is trained. The results of the DCNN model have shown the AUC of over 80% and the 80% Sen. Fotin et al. [32] have developed a CAD framework of the DBT mass detection using a DCNN that is trained on the generated candidate region of interest (ROIs), which contains 1864 breast lesions in the mammography and 339 breast lesions from the DBT images data. It is reported that their model achieved an Acc of 86.40% and 89% Sen. The latent bilateral feature representations of masses in reconstructed DBT volumes *o* are classified with the DCNN model proposed by Kim et al. [31], in which low-level features are extracted from the ROIs and the corresponding ROIs through the convolutional layers separately. To represent the high-level bilateral features of the DBT masses, the low-level features are combined in the fully connected layer. The AUC of 0.847 for the latent bilateral feature representation model is reported. Concerning our method, we obtained 0.871 Acc, 0.869 Sen, 0.882 Spe, and an AUC of 0.859 for the testing dataset with 87 DBT volumes.

In other models, not based on DCNN, we select the works of Chan et al. [15], Palma et al. [18], and Schie et al. [17]. Chan et al. [15] introduce three methods based on 2D and 3D, and the hybrid that combines 2D and 3D. For the hybrid method, they report 80% Sen with 1.23 FPs per volume for the dataset of 100 DBT images containing 69 malignant patient cases. Palma et al. [18] have developed the two-channel DBT masses detection CAD framework, in which every channel classifies one type of DBT lesions. They combine the findings from channels with the disjunctive fusion method. Their results show 90% Sen for 101 DBT volumes containing 53 lesions. van Schie et al. [17] present a two-stage method. The ROIs in 2D slice images are detected in the first step, and then extracted regions from 2D slice images are combined to localize 3D ROIs on DBT volumes in the second step. Obtained results on the DBT images data from 192 patients with 49 patients having one or more malignancies show 80% Sen with 3 FPs per volume. Reiser et al. [34] introduce the approach that detects DBT masses in 2D projection views then using the visibility angular range of findings combines the detections, and 90% Sen for 36 DBT volumes is reported.

**Figure 5:**
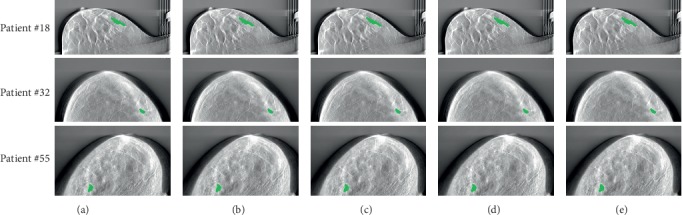
Examples of DBT masses segmented by our U-Net architecture and other classical CAD frameworks. (a) Proposed U-Net. (b) Reiser et al. (c) Kim et al. (d) Fotin et al. (e) Samala et al.

Figure 5 shows examples of DBT masses segmented by our U-Net architecture and other classical CAD frameworks. However, it is not a feasible to make a fair comparison between our CAD model with other models on DBT images because other models are trained and tested on different private datasets that are not public. Although the proposed DBT mass automatic segmentation CAD model could not achieve the best overall segmentation performance, our U-Net architecture achieves the 87.1% Acc and 86.9% Sen with an AUC of 0.859. The experimental results show that our approach achieves promising results given the fact they are obtained on DBT images data, and the U-Net model is trained on 2D slice images from DBT volumes and not on 2D mammography dataset. Although the proposed CAD framework has achieved promising results in the automatic segmentation of DBT masses, it can be further improved when more DBT images data are available. The main limitation of this work is the lack of sufficient DBT image data. To achieve satisfactory general performance, the proposed CAD framework requires diverse data and more structural distortion samples. We intend to identify all early signs of breast lesions in DBT images based on the method we used in future; by automatically detecting lesions in the DBT image, physicians can make diagnosis more accurately and quickly and surgeons can rely on it to discuss the procedure with colleagues.

## 4. Conclusions

Our study presents a novel U-Net architecture-based CAD model for the automatic detection of masses in DBT slice images, and we compare this model with other classical CAD frameworks. The advantages of the proposed U-Net architecture are that the U-Net model shortcuts among different layers can provide both global and local structural information of input images for breast mass detection. We demonstrate that our proposed U-Net CAD framework achieves promising results in the automatic segmentation of the DBT masses and exhibits outperformance compared with other classical CAD frameworks using the metrics of accuracy, AUC, specificity, and sensitivity. The future research work will focus on the combination of 3D reconstruction image information and 2D efficient data information. This combination is expected to improve the accuracy of other early signs of breast cancer detection procedures, which will be especially valuable when more clinical cases are available.

## Figures and Tables

**Table 1:** Accuracy obtained using various voting schemes.

Voting scheme	Train on T1, test on T1	Train on T2, test on T2
Majority voting	0.85 ± 0.021	0.86 ± 0.018
Maximum probability	0.87 ± 0.017	0.89 ± 0.009
Sum of probability	0.86 ± 0.013	0.87 ± 0.012
Connectivity	0.85 ± 0.011	0.86 ± 0.017

**Table 2:** Sensitivity (Sen) of different types of train-test combinations using 5-fold crossvalidation.

Train	Test on H1	Test on H2
Same hospital	0.88 ± 0.009	0.89 ± 0.021
Different hospitals	0.78 ± 0.015	0.86 ± 0.013
Both hospitals	0.87 ± 0.011	0.88 ± 0.015

**Table 3:** Specificity (Spe) of different types of train-test combinations using 5-fold crossvalidation.

Train	Test on H1	Test on H2
Same hospital	0.89 ± 0.011	0.89 ± 0.017
Different hospitals	0.86 ± 0.009	0.87 ± 0.013
Both hospitals	0.88 ± 0.013	0.88 ± 0.021

**Table 4:** Accuracy (Acc) of different types of train-test combinations using 5-fold crossvalidation.

Train	Test on H1	Test on H2
Same hospital	0.88 ± 0.011	0.89 ± 0.009
Different hospitals	0.85 ± 0.019	0.79 ± 0.015
Both hospitals	0.86 ± 0.021	0.87 ± 0.019

**Table 5:** Comparisons of selected studies in the detection of masses in the DBT images.

Method	Classifier	DBT dataset size	Sen	Acc	AUC
Kim et al. [31]	LDA	36	0.90	—	—
Shamsolmoali et al. [28]	SVM	160	—	—	0.847
Sajjad et al. [29]	DCNN	344	0.89	0.864	—
Glorot and Bengio et al. [30]	DCNN	324	0.80	—	0.80
Palma et al. [17]	SVM	101	0.90	—	—
Chan et al. [16]	Neural network	752	0.80	—	—
Reiser et al. [14]	LDA	100	0.80	—	—
Proposed	U-net	87	0.869	0.871	0.859

## Data Availability

The raw/processed data required to reproduce these findings cannot be shared at this time as the data also forms part of an ongoing study.
